# RETRACTION: Hydrogen Sulfide Is a Regulator of Hemoglobin Oxygen-Carrying Capacity via Controlling 2,3-BPG Production in Erythrocytes

**DOI:** 10.1155/omcl/9892050

**Published:** 2025-08-13

**Authors:** Oxidative Medicine and Cellular Longevity

RETRACTION: G. Wang, Y. Huang, N. Zhang, et al., “Hydrogen Sulfide Is a Regulator of Hemoglobin Oxygen-Carrying Capacity via Controlling 2,3-BPG Production in Erythrocytes,” *Oxidative Medicine and Cellular Longevity* 2021 (2021): 8877691, https://doi.org/10.1155/2021/8877691.

The above article, published online on 13 February 2021 in Wiley Online Library (wileyonlinelibrary.com), has been retracted following the agreement of the journal's Chief Editor Jeannette Vasquez-Vivar; and John Wiley & Sons Ltd.

The retraction has been agreed following an investigation undertaken by the publisher, where unexpected similarities were identified between the images of BPGM-stained erythrocytes of WT and Cse−/− mice in Figure 4c, and Human erythrocytes without GYH in Figure 5e. The authors were asked to provide raw data, however they were unable to do so.

As a result of the investigation, the data of this article are considered unreliable.

The authors disagree with this retraction.

